# Pin-Shaped Surface Structures Generated by Laser Single Pulse Drilling for High-Strength Interfaces in Thermally Joined Polymer–Metal Hybrids

**DOI:** 10.3390/ma16020687

**Published:** 2023-01-10

**Authors:** Erik Saborowski, Philipp Steinert, Thomas Lindner, Andreas Schubert, Thomas Lampke

**Affiliations:** 1 Materials and Surface Engineering Group, Faculty of Mechanical Engineering, Chemnitz University of Technology, D-09107 Chemnitz, Germany; 2 Micromanufacturing Technology Group, Faculty of Mechanical Engineering, Chemnitz University of Technology, D-09107 Chemnitz, Germany

**Keywords:** polymer–metal hybrid, laser micromachining, polyamide 6, EN AW-6082, mechanical interlocking, surface structuring, thermal joining

## Abstract

Laser structuring is by far the most investigated metal surface-pretreatment method for creating adhesion in polymer–metal hybrids. Especially, cone-like protrusions show excellent wetting behaviour as well as high compound strength. However, the processing time is extremely high. Therefore, this paper assesses a process strategy for creating pin structures with scalable height by single pulse drilling with an Nd/YVO_4_ nanosecond laser system on EN AW-6082 aluminium alloy. The strength testing is carried out by butt-bonded hollow cylinder torsion. The samples are manufactured by heat-conduction thermal joining with polyamide 6. Ten different surface structures with two different ablation diameters are investigated and compared to cone-like protrusions in terms of processing time, wetting behaviour, shear strength and fracture behaviour. The experimental results show that pulse drilling pins structures with high aspect ratio reach-strength values close to cone-like protrusions but with 31 times higher processing rate.

## 1. Introduction

**Figure 1:**
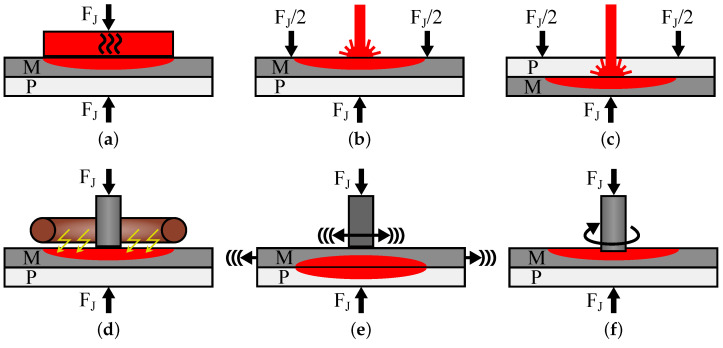
Schematic illustration of different thermal joining processes (P = polymer, M = metal): (**a**) heat conduction joining, (**b**) laser-heat conduction joining, (**c**) laser direct joining, (**d**) induction joining, (**e**) ultrasonic welding, (**f**) friction welding.

Polymer–metal hybrids (PMH) offer great potential for lightweight applications. Advantages in pure metal applications are, e.g., higher specific strength or stiffness. Thus, they are used as sandwich structures in automotive applications such as hoods [1] and top floor panels [2]. As fibre-metal laminates (FML), they are also used in aircraft components with high safety requirements such as fuselages and wings, due to their excellent damage tolerance [3]. In this context, thermal joining in combination with micro-mechanical interlocking is a promising technology for connecting metals to thermoplastic polymers. Thereby, the joining partners are pressed together by a joining force $F_{J}$, while heat is transferred either into the metallic joining partner or directly into the joining zone between both materials. The polymer melts and wets the metal surface. After solidification, it interlocks the metal surface microstructure, thus creating a strong bonding. The thermal energy is transferred to the metal, e.g., by a heated stamp (Figure 1a) [4,5,6,7,8], a laser beam (Figure 1b) [9,10,11,12,13,14,15,16,17,18], an induction coil (Figure 1d) [19,20,21,22] or a rotating pin (Figure 1f) [23,24,25,26,27]. Techniques for creating the heat directly in the joining zone are direct laser joining using polymers with sufficient transparency (Figure 1c) [9,28] or ultrasonic welding (Figure 1e) [8,29,30,31,32]. Next to thermal joining, in-mould assembly is another technology for manufacturing micro-mechanically interlocked PMHs. Thereby, a metallic insert is placed into an injection moulding machine and directly overmoulded with polymer [33,34,35,36,37,38].

The metal surface structure has the highest impact on the compound strength of micro-mechanically interlocked PMHs. The real surface area determines the number of contact points for the formation of different bonding forces in the interface [39]. For, e.g., polyamides, hydrogen bonds [40] or even covalent bonds [41] are described. Undercuts interlock the polymer and prevent both joining partners from separation when a load is applied. Thus, highly undercut surface structures provide a significantly higher compound strength than slightly undercut ones [37]. Sub-structures provide additional surface area and undercuts. They are, e.g., formed from solidified melt during laser structuring [15,21,35,42]. The structure density describes the structured proportion of the projected surface area. Various authors have shown that reducing the distance between groove-, crater- or pin-shaped profile elements and the associated increase in structure density increases the compound strength [15,16,21,28,32,35,42,43]. Furthermore, the aspect ratio describes the ratio of height (or depth) to width of a profile element. An increase in crater or groove depth and the associated increase in aspect ratio caused partly an increase [15,42,44,45], partly an increase with stagnation above a certain depth [9,15,35,46] or partly a complete stagnation [9] of the compound strength in recent studies. Both high structure density and aspect ratio increase the real surface area, the amount of undercuts as well as the possibility of the formation of sub-structures.

**Figure 2:**
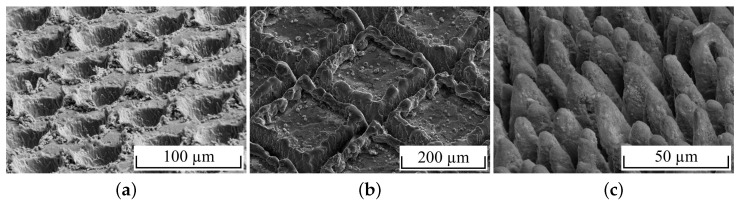
Topography images of laser structured surfaces with different processing strategies: (**a**) crater, (**b**) groove [4], (**c**) CLP [5].

For surface pretreatment, mechanical blasting with, e.g., alumina is an industrially established, low-cost process for creating stochastically arranged, rugged structures that can achieve acceptable adhesion [4,5,6,8,12,20,34,47,48,49]. However, laser structuring is by far the most investigated pretreatment method. Thereby, material is removed from the metal surface by pulsed laser radiation with high power density. The high energy input causes rapid heating of the surface, which abruptly vaporises material. The achievable roughness, ablation depth and the resulting surface structure are determined by the process parameters power, pulse repetition frequency and pulse spacing, as well as the processing strategy [50]. Single pulse drilling is conducted to create crater-shaped ablations. Depending on the arrangement of the ablations, the resulting surface structure consists of single craters [8,9,28,35] (Figure 2a) or grooves [4,9,10,11,12,13,14,15,16,21,24,28,32,36,38] (Figure 2b). The wetting with molten polymer is often insufficient for single craters, since degassing of entrapped air is not possible [8,9,35]. Grooves are produced by tightly overlapping single ablations along a line or a grid. Since degassing is possible along the grooves, a better wetting behaviour than for single craters is achieved [9]. A second processing strategy is the formation of cone-like protrusions (CLP, Figure 2c) using spatially overlapped pulses of a laser beam. The nano- or micrometer-sized protrusions with high aspect ratio are densely arranged [8,17,51,52]. Advantages of CLPs are good wetting behaviour as well as the high achievable compound strength, which leads to almost pure cohesive fracture during strength testing [8,17]. The disadvantage is the slower processing rate compared to single pulse drilling due to the greater energy density required.

The aim of this contribution is the assessment of pin structures created by single pulse drilling, thus combining high structure density, fast processing rate and good wetting behaviour. To this end, the following points are hypothesized:

1. Laser single pulse drilling with slightly overlapping ablations creates a surface structure formed from pin-shaped profile elements.
2. The resulting surface structure shows a good wetting behaviour comparable to CLP structures.
3. The number of scans and the aspect ratio of the profile elements are proportional.
4. The ratio of compound strength to processing rate and the aspect ratio of the profile elements are proportional.

The experiments are carried out on an EN AW-6082/PA6 PMH. In total, 10 different pin surface structures with two different ablation diameters are applied on the aluminium surfaces. All obtained results are compared to CLP structures and structures created by mechanical blasting, which are deduced from our previous investigations presented in [5,6]. The samples are produced by heat conduction hot pressing. The shear strength is determined using a butt-bonded hollow cylinder torsion test that ensures a homogeneous shear stress distribution in the interface between both joining partners during testing [53,54]. The wetting behaviour is assessed by cross-sectional images of the joined samples. Finally, a fracture surface analysis of all tested samples is performed.

## 2. Materials and Methods

### 2.1. Materials

The surface structuring was conducted on the face of hollow cylinders made up of EN AW-6082 (3.2315) aluminium alloy (AMCO Metall-Service GmbH, Bremen, Germany). The polymeric joining partner was made up of PA6 (Technoplast v. Treskow GmbH, Lahnstein, Germany). The supplier specifications are shown in Table 1. Since the mechanical properties of the PA6 strongly depend on the moisture content, conditioning according to DIN ISO 1110 at 70 °C and 62% humidity was conducted for 3 days before testing.

### 2.2. Surface Structuring

The laser structuring process was conducted by a Nd:YVO_4_ nanosecond laser system from Spectra Physics^®^ (Santa Clara, USA) with a telecentric F-Theta lens (focal length = 80 mm). The parameters of the laser system are shown in Table 2. Multiple, line-wise scanning of the aluminium surface area with overlapping single pulses was performed for the realization of the surface microstructures. The processing parameters are shown in Table 3.

Stochastically distributed CLP structures were manufactured in accordance with [8,51]. Thereby, a defined energy input of 3–6 J·cm${}^{-2}$ was applied to the surface by a defocused laser spot, measuring about 55 µm in diameter.

**Figure 3:**
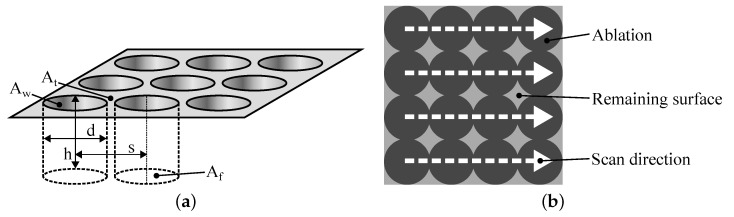
Single pulse drilling strategy: (**a**) schematic illustration of a drilling surface structure, (**b**) strategy for creating pin structures.

Deterministically distributed pin structures were manufactured by single pulse drilling. Figure 3a shows the scheme of a metal surface structured by cylindrical craters, where *d* denotes the crater diameter, *h* the crater height and *s* the crater spacing. According to [39], chemical and physical bonding forces at the interface of PMHs are proportional to the real surface area of the metal $A_{r}$. In order to maximize the bonding forces within a given projected area $s^{2}$, the ratio $A_{r}\cdot s^{-2}$ has to be maximized. Assuming a perfect cylindrical shape of the craters, $A_{r}=A_{T}+A_{W}+A_{F}$ within $s^{2}$. $A_{T}$ denotes the unstructured top area of the metal surface, $A_{F}$ the floor area of the crater and $A_{W}$ the wall area of the crater. For a perfect cylindrical shape, $A_{T}+A_{F}=s^{2}$ and $A_{W}=\pi \cdot d\cdot h$. Hence, $A_{r}\cdot s^{-2}=\left(s^{2}+\pi \cdot d\cdot h\right)\cdot s^{-2}$. In order to maximize $A_{r}\cdot s^{-2}$, *s* has to be equal to *d*, which sets the distance between two adjacent craters to 0. However, this presumably would cause air pockets in the resulting PMH, as described in [8,9,35]. Therefore, the wall between the craters is perforated by setting s<d to enable degassing during the joining process. Since the perforation reduces $A_{W}$, the difference between *s* and *d* should be as low as possible. Thus, nearly the maximum $A_{r}\cdot s^{-2}$ for a given ablation volume is achieved. Two different crater diameters (approx. 14 µm and 45 µm) were realized by varying the focus positions (0 mm and +0.5 mm). The pulse spacing was 12.5 µm for the smaller crater diameter (L12) and 40 µm for the higher crater diameter (L40). The final surface structure consists of pin-shaped profile elements distributed in a grid where the height of the pins is adjusted by the number of scans. Figure 3b illustrates the realized processing strategy schematically.

For producing the mechanically blasted reference samples (MB), alumina particles with F16 grit size (particle size: 1000–1400 µm) were applied to the aluminium surface with a blasting distance of 100 mm, a blasting angle of 75°, a blasting pressure of 0.2 MPa and a treatment time of 10 s.

### 2.3. Sample Production and Testing

**Figure 4:**
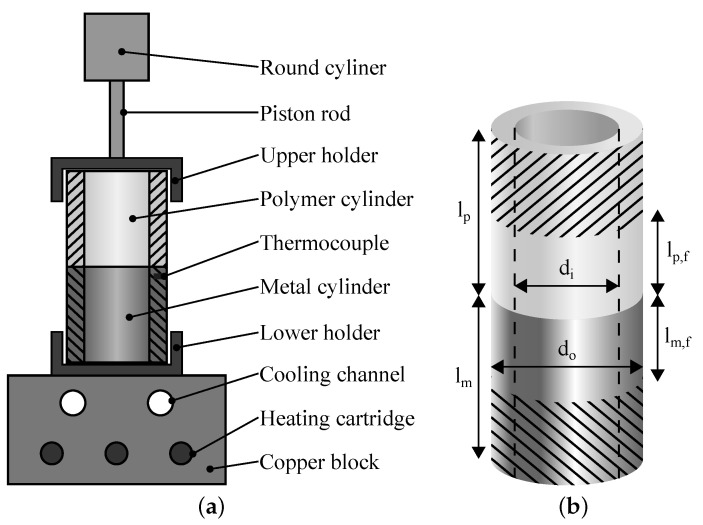
Hollow cylinder sample: (**a**) heat conduction hot pressing tool, (**b**) geometry.

The hollow cylinder samples were produced with a heat conduction hot pressing process. Figure 4a illustrates the joining tool. Beforehand, aluminium and polymer components were ultrasonically degreased in ethanol and the PA6 cylinders were dried for 7 days at 70 °C. The hot pressing process was conducted with an isobaric joining pressure of 0.2 MPa at the interface between the joining partners. The temperature in the interface region was measured by a thermocouple placed inside a drill hole slightly below the metal surface. The copper block was heated for approx. 330 s until the polymer on top of the metal melted at an interface temperature of 240 °C. Afterwards, air-cooling of the copper block was activated for approx. 500 s till the interface temperature fell below 100 °C. Finally, joining pressure and sample were removed from the joining-tool. The samples were reworked by turning after the joining process to ensure the necessary concentricity for the torsion test. Four samples of each structure were produced and tested.

Figure 4b illustrates the geometry of the hollow cylinder samples. The outer diameter $d_{o}$ = 28 mm, the inner diameter $d_{i}$ = 23 mm, the length of the metal cylinder $l_{m}$ = 40 mm and the length of the polymer cylinder $l_{p}$ = 60 mm. A PTT 250 K1 hydraulic testing machine (Carl Schenck AG, Darmstadt, Germany) was used for the torsion test. The samples were clamped with ER40-472E collets according to ISO 15488. A steel plug was inserted into the polymer cylinder in order to prevent it from yielding when the collet was tightened. The unclamped length of the metal part during testing $l_{m,f}$ = 20 mm. For the polymer part, an unclamped length $l_{m,p}$ of 30 mm was used for the MB samples, while only 10 mm were used for the laser-structured samples. $l_{m,p}$ had to be reduced for the samples with higher strength, since the maximum twist angle of the testing machine (90°) would have been exceeded otherwise. The angular velocity of the testing machine was 15°/min for the MB samples and 5°/min for the laser-structured samples. Thus, a shear rate of 2·10^−4^·s^−1^ in the polymer was achieved in both cases. The deformation of the metal part was neglected, hereby, since it is approx. 39 times stiffer than the polymer, according to Table 1. The shear strength of the interface $\tau_{max}$ was calculated by Equation (1), where $T_{max}$ denotes the maximum torque during the torsion test.

(1) $$\tau_{max}=\frac{16\cdot T_{max}d_{a}}{\pi \left(d_{a}^{4}-d_{i}^{4}\right)}$$

### 2.4. Surface Characterization

Roughness measurements were carried out using a Hommel-Etamic^®^ T8000 stylus profiler (JENOPTIK AG, Jena, Germany). Five measurements with an evaluation length of 12.5 mm were recorded and evaluated for each surface structuring process. Thereby, a 2 μm/60° stylus tip was used for capturing the highest possible number of profile details. The arithmetical mean height Ra and the average maximum profile height Rz were calculated in accordance to ISO 4287. Since both values tend to underestimate the actual roughness for tightly spaced profile elements [5,6], the structure height SH was determined, additionally, as alternative to Rz. SH, which describes the vertical extent of the surface structure, was obtained from 3 images of 5 cross-sections (15 images in total) for each sample. Thereby, the distance between the highest and the lowest point of the profile line $SH_{i}$ was measured with the image evaluation software ImageJ 1.52a [55]. SH was then calculated from the average of all individual measurements (Equation (2)).

(2) $$SH=\frac{1}{15}\sum_{i=1}^{15}SH_{i}$$

Since the distances between the ablations and the ablation diameters are assumed to stay nearly constant regardless of the number of scans, SH is considered as representative measure of the aspect ratio. Consequently, SH is used to evaluate the working hypotheses.

## 3. Results

### 3.1. Surface Characteristics, Wetting Behaviour and Shear Strength

**Figure 5:**
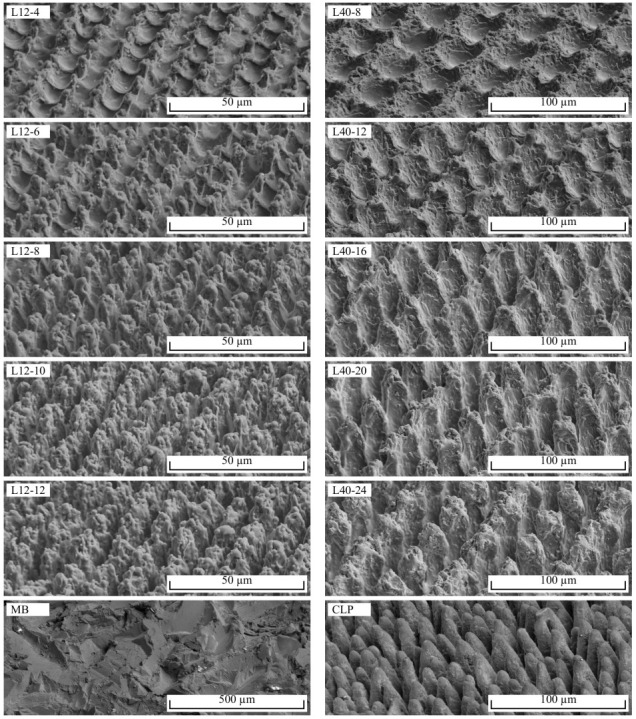
SEM images of all investigated surface structures, stage inclined at an angle of 60°.

**Figure 6:**
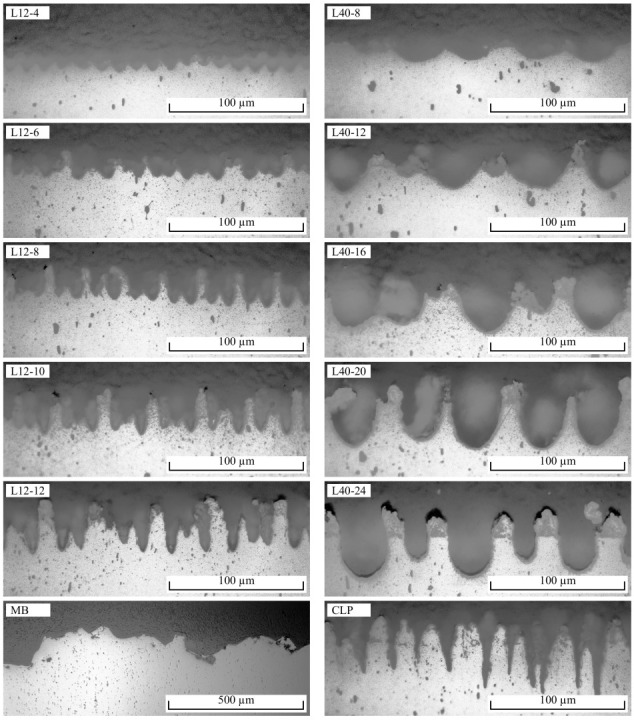
Optical microscopy images of the cross-sections of all joined samples.

Table 4 shows $R_{a}$, $R_{z}$, SH and $\tau_{max}$ for all investigated samples, whereby SD denotes the standard deviation. Note that the roughness values $R_{a}$ and $R_{z}$ stagnate from L12-10 to L12-12 as well as from L40-16 to L40-24, despite an increasing SH. This is due to incomplete penetration of the tightly spaced ablations by the tip of the stylus profiler, as described in [5,6]. Consequently, the measured values do not represent well the actual roughness profile and are, therefore, not discussed further. Figure 5 illustrates secondary electron microscopy (SEM) images of the surface topographie. Figure 6 shows the corresponding cross-sectional images as well as the wetting between aluminium and PA6.

**Figure 7:**
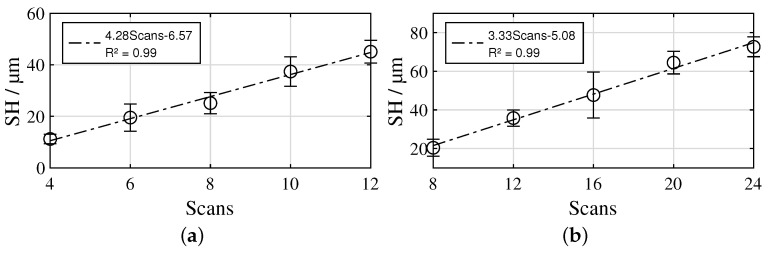
Correlation between the number of scans and SH: (**a**) L12 structures, (**b**) L40 structures; mean values ± 1SD.

In the case of the L12 samples, deterministically distributed pins with SH of 11–45 µm are formed. SH increases monotonically with the number of scans (Figure 7a, $R^{2}$ = 0.99). The bottoms of the ablations show a relatively smooth surface, while the surface of the pins between the ablation craters consists of randomly arranged, rugged, solidified melt. As the number of scans increases, a thickening of the pins is observed in addition to an increase in SH. In the case of the L40 samples, SH is in the range of 20–73 µm. SH also increases monotonically with the number of scans (Figure 7b, $R^{2}$ = 0.99). L40-8- and L40-12-structures are formed from ablations with intervening ridges, on which solidified melt agglomerates. From 16 scans, more pin-shaped, tapered profile elements are formed. With 20 or 24 scans, the profile elements clearly form pins. The cross-sectional images in Figure 6 show that the tips of the profile elements consist of solidified melt that is not completely bonded to the base material. The CLP structure is characterized by stochastically distributed, steeply and conically shaped pins with SH of 71 µm and about 18 µm spacing. The MB structure has the highest SH of 101 µm of all the surface structures studied. The surface is rugged and has many sharp edges.

In order to make air inclusions more visible, the colours in Figure 6 were modified using the Equalize function of the GNU Image Manipulation Program 2.10.28. For the pulse-drilling structures, air inclusions are not visible in 9 out of 10 cases. Only the L40-24 structure shows slight air inclusions and delamination in the bottom of the ablations. CLP and MB structures also show no air inclusions.

**Figure 8:**
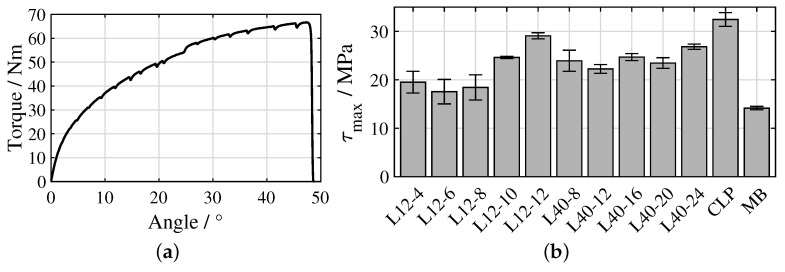
Results of the hollow cylinder torsion tests: (**a**) exemplary angle/torque curve of the L12-12 structure, (**b**) $\tau_{max}$ of all investigated structures; mean values ± 1SD.

**Figure 9:**
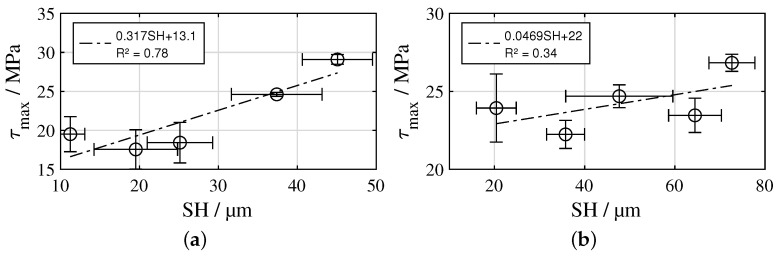
Correlation between SH and $\tau_{max}$: (**a**) L12 structures, (**b**) L40 structures; mean values ± 1SD.

An exemplary angle/torque curve of the hollow cylinder torsion test is illustrated in Figure 8a, showing a decrease in stiffness with increasing load due to viscoelastic softening of the PA6. Figure 8b illustrates $\tau_{max}$ for all investigated samples. For the L12 structures, a stagnation of $\tau_{max}$ in a range of 17.6–19.5 MPa can be seen from L12-4 to L12-8, while from L12-8 to L12-10, as well as from L12-10 to L12-12, there is a clear increase to 24.6 MPa and 29.1 MPa, respectively. SH and $\tau_{max}$ show moderate correlation with $R^{2}$ = 0.78 (Figure 9a). In the case of the L40 structures, $\tau_{max}$ from L40-8 to L40-20 varies randomly in a small range of 22.2–24.7 MPa, only in the case of the L40-24 structures is there a moderate increase to 26.8 MPa. SH and $\tau_{max}$ show only low correlation with $R^{2}$ = 0.34 (Figure 9b). The CLP structure reached the highest $\tau_{max}$ of 32.5 MPa, whereas the MB structure reached the lowest with 14.2 MPa.

### 3.2. Fracture Analysis

**Figure 10:**
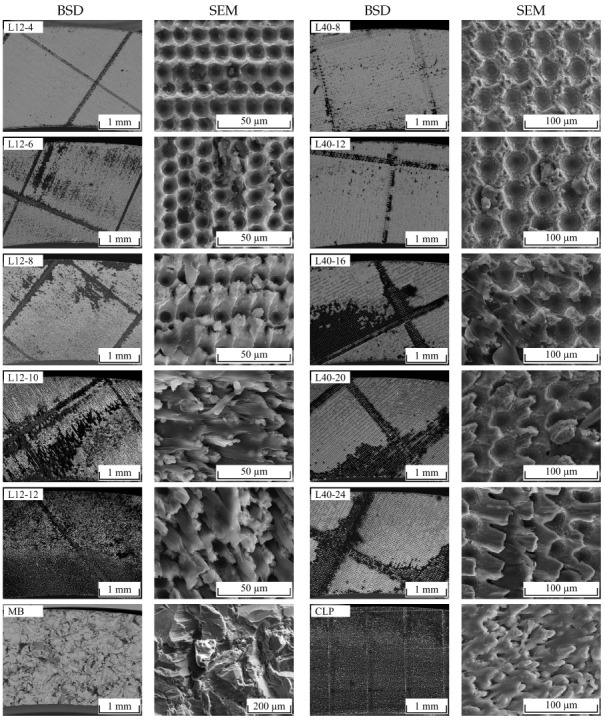
BSD (**left**) and SEM (**right**) images of the fractured metal surfaces of all investigated surface structures.

Figure 10 illustrates the fracture surfaces of all investigated samples. Dark areas indicate fractured polymer as the images were recorded using a back scattering detector (BSD) that allows for imaging material contrast. Additionally, SEM images with high magnification are shown for illustrating the exact fracture behaviour.

In the case of the L12 samples, an increasing proportion of cohesively fractured polymer can be seen with increasing numbers of scans. The L12-4 structure shows only very isolated polymer residues in the ablations. Partial bending of the pins between the ablations is visible. The L12-6 structure already shows a higher proportion of cohesively broken polymer and also partially bent pins. From the L12-8 structure onwards, the load direction is clearly visible in the orientation of the bent pins. In these cases, almost all pins are bent and the proportion of cohesively fractured polymer increases further. In the L12-10 structure, significant portions of the fracture surface are covered with polymer residues. This is held between the pins under load until they bend over and the polymer finally breaks. In the L12-12 structure, the fracture surface is almost completely covered with polymer residues from which the plastically deformed pins protrude.

In the case of the L40 samples, the fracture surfaces in the BSD images show only small amounts of polymer residues for each of the L40-8 and L40-12 structures, while L40-16, L40-20 and L40-24 structures each show a comparable amount of polymer-covered area. In the SE images, however, polymer residues can be identified on the ridges between the ablations, especially in the L40-8 structure. The polymer adheres to the ridges in the substructures and breaks cohesively in these areas when loaded. In the L40-12 structure, this effect remains in a reduced form. From the L40-16 structure onwards, more pin-shaped profile elements form between the ablations, which bend over under shear load. In some places, entire areas of the polymer are broken, while in other places hardly any polymer residues are visible. From the L40-20 structure onwards, the direction of loading is clearly recognisable from the orientation of the bent pins. As with the L40-16 structure, entire areas of the polymer are broken out in some places, while hardly any polymer residues are visible elsewhere. The fracture surface of the L40-24 structure is characterised by bent pins with torn-off tips. The tip, consisting of solidified melt, is barely connected to the base material, as can be seen in Figure 6, and breaks under load. Polymer residues are partially visible between the bent pins. A considerable portion of the surface is covered with completely torn out polymer.

For the MB structure, only a small amount of polymer residue covers the fracture surface. For the CLP structure, the complete surface is covered with fractured polymer, from which plastically deformed pins protrude. Thus, the fracture behaviour is comparable to the L12-12 structure.

## 4. Discussion

With reference to the working hypotheses, the obtained results for the L12 structures are interpreted as follows: In each case, the laser structuring process produces a completely remelted surface, which, thus, already exhibits a sub-roughness deviating from the untreated metal surface in all areas at a low number of 4 scans. This already leads to an acceptable shear strength. Increasing the number of scans to 6 or 8 results in an increase in the height of the pin-shaped profile elements and, thus, an increase in SH. However, when loaded, the pins bend over rapidly without initially causing an increase in shear strength. A further increase in the number of scans to 10 or 12 results in a further thickening of the pins due to additional available melt, so that the bending of the pins only occurs at higher loads. Consequently, the correlation between SH and $\tau_{max}$ is only moderate (Figure 9a) and Hypothesis 4 is only partially confirmed. However, Hypotheses 1, 2 and 3 are confirmed completely since pin structures are created in every case, the wetting behaviour with PA6 is comparable to the CLP samples and SH highly correlates with the number of scans (Figure 7a).

For the L40 structures, the results are interpreted as follows: As with the L12 structures, a completely remelted surface is produced in each case. In the case of the L40-8 structure, a strongly rugged substructure forms on the ridges between the ablations, which takes up large portions of the metal surface. In these areas, the polymer fractures cohesively, so that a significant contribution to $\tau_{max}$ is assumed. However, this rugged substructure does not significantly contribute to SH. The described effect decreases with an increasing number of scans, since more pin-shaped structures are formed. The formation of the pins leads on the one hand to an increase in SH and on the other hand to a reduction of the parts of the metal surface covered with rugged, solidified melt. With respect to $\tau_{max}$, the supporting effect by the pins increases with increasing number of scans, while that of the rugged substructure between the ablations decreases. Additionally, the tip of the pins tends to tear off more as the number of scans increases. These effects cancel each other out, so that a stagnation of $\tau_{max}$ occurs. Only with the L40-24 structure is the strength moderately increased. Thus, the correlation between SH and $\tau_{max}$ is low (Figure 9b) and Hypothesis 4 is not confirmed. Hypothesis 1 is confirmed partially, since 16–24 scans created pin-shaped profile elements, whereas 8–12 scans created more crater-shaped ablations. Hypothesis 2 is confirmed for 8–20 scans, whereas air inclusions occur in the interface of the L40-24 samples. However, Hypothesis 3 is confirmed completely since SH highly correlates with the number of scans (Figure 7b).

The L12-12 structure achieved 89.5 % of the $\tau_{max}$ of the CLP structure (L12-12: 29.1 MPa; CLP: 32.5 MPa) with 31 times higher processing rate (L12-12: 1.56 cm·min${}^{-1}$; CLP: 0.05 cm·min${}^{-1}$, s. Table 3). The ratio of the distance to the height of the profile elements is approximately the same (L12-12: 12.5/45; CLP: 18/71). However, since there is no distance between the bases of the pins of the CLP structure, they have a conical shape and are, thus, more stable. In the L12-12 structure, on the other hand, the distance between the bases corresponds to the ablation diameter. Consequently, the pins have a more straight shape and bend at lower loads. With reference to the increasing trend between SH and $\tau_{max}$ in Figure 9a, it is assumed that a higher $\tau_{max}$ will be achieved with a larger number of scans. However, it can also be assumed that the shear strength will stagnate above a certain number of scans, as then, similar to the CLP structure, almost pure cohesive fracture of the polymer may occur. The limits of the achievable shear strength will be the subject of future work. Furthermore, it will be an aim to modify the CLP structures in terms of their geometric characteristics and arrangement by adapting the processing conditions and using additional process energies. The potential of these measures with regard to process reliability and processing speed will also be considered in future work.

## Figures and Tables

**Table 1:** Supplier specifications of the used materials, PA6 in moist condition according to ISO 1110.

	EN AW-6082	PA6
Density/kg·m${}^{-3}$	2700	1140
Young’s modulus/MPa	70,000	1800
Yield strength/-stress/MPa	240–320	60
Ultimate strength/MPa	300–350	-
Elongation to failure/%	8–14	200
Melting temperature/°C	660	223
Thermal expansion coefficient/10${}^{-6}$·K${}^{-1}$	23.4	70

**Table 2:** Parameters of the used laser system.

Wave Length	Pulse Duration	Repetition Rate	Max. Avg. Power	Beam Quality M${}^{2}$	Beam Shape
532 nm	10 ns	15–300 KHz	13 W	<1.3	gaussian

**Table 3:** Used processing parameters for the laser structuring processes.

Structure	Pulse Frequency/kHz	Pulse Distance/μm	Focus Change/mm	Scans	Processing Rate/cm${}^{2}\cdot$min${}^{-1}$
CLP	100	2.0	+1.0	5	0.05
L12-4	200	12.5	0.0	4	4.69
L12-6	200	12.5	0.0	6	3.13
L12-8	200	12.5	0.0	8	2.34
L12-10	200	12.5	0.0	10	1.88
L12-12	200	12.5	0.0	12	1.56
L40-8	25	40.0	+0.5	8	3.00
L40-12	25	40.0	+0.5	12	2.00
L40-16	25	40.0	+0.5	16	1.50
L40-20	25	40.0	+0.5	20	1.20
L40-24	25	40.0	+0.5	24	1.00

**Table 4:** Surface characteristics and shear strength of all investigated samples, mean values ± 1SD.

Structure	Ra/μm	Rz/μm	SH/μm	$\tau_{\max}$/MPa
L12-4	1.93 ± 0.25	14.6 ± 1.8	11.2 ± 1.8	19.5 ± 2.3
L12-6	2.71 ± 0.23	19.0 ± 1.5	19.5 ± 5.3	17.6 ± 2.5
L12-8	2.78 ± 0.25	19.5 ± 1.4	25.1 ± 4.1	18.4 ± 2.6
L12-10	4.25 ± 0.20	27.0 ± 1.9	37.4 ± 5.7	24.6 ± 0.2
L12-12	3.63 ± 0.15	24.9 ± 1.6	45.1 ± 4.4	29.1 ± 0.6
L40-8	4.49 ± 0.87	30.9 ± 3.4	20.4 ± 4.4	23.9 ± 2.2
L40-12	6.40 ± 0.73	39.6 ± 4.8	35.8 ± 4.2	22.2 ± 0.9
L40-16	7.20 ± 0.67	44.4 ± 4.8	47.7 ± 11.9	24.7 ± 0.7
L40-20	7.58 ± 0.44	47.4 ± 3.1	64.5 ± 5.9	23.5 ± 1.1
L40-24	7.01 ± 0.85	42.7 ± 6.4	72.6 ± 5.1	26.8 ± 0.5
CLP	5.59 ± 0.17	45.6 ± 1.2	70.9 ± 6.6	32.5 ± 1.4
MB	21.98 ± 2.43	130.8 ± 8.0	101.2 ± 24.4	14.2 ± 0.4

## Data Availability

The authors confirm that the data to support the findings of this study are available within the article.

## Abbreviations

The following abbreviations are used in this manuscript:

BSD	Back scattering detector
CLP	Cone-like protrusions
FML	Fibre metal laminate
PMH	Polymer–metal hybrids
PA6	Polyamide 6
SD	Standard deviation
SEM	Secondary electron microscopy
